# A microRNA binding site polymorphism in the 3′ UTR region of VEGF-A gene modifies colorectal cancer risk based on ethnicity: a meta-analysis

**DOI:** 10.1186/s43046-022-00118-3

**Published:** 2022-04-25

**Authors:** Sai Sushmitha Kontham, Charles Emmanuel Jebaraj Walter, Zioni Sangeetha Shankaran, Arvind Ramanathan, Nirmala Karuppasamy, Thanka Johnson

**Affiliations:** 1 Department of Biotechnology, Sri Ramachandra Institute of Higher Education & Research (formerly Sri Ramachandra Medical College & Research Institute), Chennai, India; 2 School of Allied Health Sciences, Sree Balaji Medical College and Hospital, Chennai, India; 3 Human Genetics Laboratory, Sree Balaji Dental College & Hospital, Bharath Institute of Higher Education & Research, Chennai, 600116 India; 4 Department of Pathology, Sri Ramachandra Institute of Higher Education & Research (formerly Sri Ramachandra Medical College & Research Institute), Chennai, India; 5 Department of Pathology, Sree Balaji Medical College and Hospital, Chennai, India

**Keywords:** VEGF-A, Colorectal cancer, +936C/T, MicroRNA polymorphisms, Meta-analysis

## Abstract

**Background:**

Vascular endothelial growth factor A (VEGF-A) plays an integral role in angiogenesis by contributing to growth, development, and metastasis of solid tumors. Recently, a single-nucleotide polymorphism +936C/T located in the VEGF-A 3′ untranslated region (UTR) facilitated the susceptibility of colorectal cancer. The association between VEGF-A gene polymorphism +936C/T and colorectal cancer risk has been widely studied in the last decade, but presently, the results furnished remain enigmatic. Hence, the study aimed to investigate the association between VEGF-A +936C/T miRNA binding site polymorphism and the risk of developing colorectal cancer.

**Methods:**

This meta-analysis included 13 published case-control studies covering 3465 cases (colorectal cancer) and 3476 healthy controls. Publication bias was examined by means of Begg’s funnel plots and Egger’s regression tests. The quality of the studies included was evaluated using Newcastle-Ottawa scale. Subgroup analyses were performed in accordance to the various ethnicities of the study subjects and the study quality.

**Results:**

From the data obtained, it is implied that VEGF-A +936C/T polymorphism did not correlate with elevated colorectal cancer risk in all genetic models. But the results acquired from the subgroup analysis in over dominant model (CT vs. CC + TT: *OR* = 1.5047, 95% *CI* = 1.19–1.90) suggest that VEGF-A +936C/T polymorphism leads to the raise in the risk of developing CRC among the East Asian population. No association was observed in Caucasian and South Asian population.

**Conclusions:**

Our results indicate that VEGF-A +936C/T polymorphism is not a risk factor for developing CRC in Caucasian and South Asian population. However, the East Asian population was related to an increased risk of developing colorectal cancer due to the presence of the minor allele.

## Background

Colorectal cancer (CRC) is currently the third most widespread cancer type and the foremost cause of cancer mortality in both men and women worldwide [1, 2]. It has been assessed that 1.4 million individuals are suffering from CRC every year, 65% of whom are from developed nations; this ailment causes about 700,000 deaths every year, and 3.5 million people continue to live with CRC [3]. It is a multifaceted disease which occurs due to the influence of numerous reasons such as environmental factors and genetic variations [4, 5], which are the root cause to considerably influence the risk of CRC [6, 7]. Angiogenesis is the process of production of new blood vessels that play a vital role in cancer development and metastases. The vascular endothelial growth factor A (VEGF-A), a heparin-binding glycoprotein, contributes to mitogenic, angiogenic, and vascular permeability activities explicit for endothelial cells. VEGF-A gene is located at chromosome 6 and comprises of eight exons [8]. VEGF-A gene belongs to the VEGF family; it encompasses four VEGF amino acid isomeric residues VEGF165, VEGF189, VEGF121, and VEGF206. This gene is extremely polymorphic, its promoter, 5′ and 3′ untranslated regions (UTRs), has a diverse range of single-nucleotide polymorphism SNPs [9]. MicroRNAs (miRNAs) are a group of single-stranded noncoding RNA molecules which regulate gene expression by binding to cognate sequences of the 3′ UTR regions of mRNAs. Their binding leads to reduction in protein translation and an increase in mRNA degradation [10]. SNPs present in the 3′ UTR targeted by miRNAs can either eliminate existing binding sites or produce illegitimate binding sites or can affect miRNA:mRNA interactions and target the mRNA expression [11]. This process results in the regulation and expression of target genes.

There are more than 15 VEGF SNPs that have been reported in diverse types of cancers [12, 13]. VEGF SNPs such as +936C > T, −2578C > A, +405C > G, -634G>C, −460C > T, and −1154G > A have been extensively studied [14]. SNPs located in the 3′ UTR region of the VEGF gene were found to be correlated with variations in VEGF protein production [15]. Among the many polymorphisms, +936C/T polymorphism located at miR-199a binding site in the VEGF-A 3′ UTR region has been demonstrated to perform a functional role. This polymorphism has been significantly associated with different types of cancer like oral, breast, colorectal cancer [16, 17], and other diseases with a recognized angiogenic basis [18].

MiR-199 is an imperative vertebrate-specific miRNA, which is associated with a wide variety of cellular and developmental mechanisms like tumor growth and progression [19]. It has been reported that MiR-199a downregulates and also suppresses tumor progression in prostate adenocarcinoma [20], chondrosarcoma [21], hepatocellular carcinoma [22], and ovarian cancer [23]. In recent times, it has been testified that miR-199a is unusually expressed in CRC [24–27]. MiR-199a plays an important role in repressing the migration and invasion of CRC cells in hypoxia-inducible factor 1-alpha/vascular endothelial growth factor (HIF-1α/VEGF) pathway and targets the discoidin domain receptor 1 [28, 29]. Shweiki et al. reported that VEGF-A production was controlled by numerous stimuli, among which hypoxia is the most significant one [30]. In hypoxic conditions, HIF-1α translocates into the nucleus and heterodimerizes with HIF-1β by binding to hypoxia-responsive elements on several genes, such as VEGF-A [31]. The underlying mechanism of miR-199a in regulation, development, and progression of CRC still remains indistinct as miR-199a has numerous target genes [32], which may be involved in tumor initiation and progression of CRC. Therefore, SNPs situated in the miRNA binding sites may affect the expression of miRNA target genes and contribute to the susceptivity of humans to develop diseases [33–36]. Thereby, it is hypothesized that SNPs present in the potential miRNA binding sites of VEGF-A gene may influence the susceptibility and progression of colorectal cancer.

Ongoing studies have demonstrated that SNPs situated in the VEGF-A gene may contribute to the development of colorectal cancer. Hence, it is commonly speculated that due to the varied ethnicities and inadequate sample size, the results of these studies remain inconclusive. Thus, this updated meta-analysis includes all eligible case-control studies which were implemented to inspect whether VEGF-A miRNA binding site polymorphism +936C/T was associated with the risk of developing colorectal cancer.

## Methods

### Literature search criteria

A systematic literature search investigating the association of VEGF-A gene polymorphism and colorectal cancer risk was performed utilizing PubMed (www.ncbi.nlm.nih.gov/pubmed), ScienceDirect (www.sciencedirect.com), and Google Scholar (www.scholar.google.com) databases to find relevant publications up to June 2020. The systematic search was conducted adopting different combinations of relevant keywords: colorectal neoplasm, polymorphism, and genetic vascular endothelial growth factor. Furthermore, the reference lists of original studies were examined manually for additional literature. All the appropriate studies, abstracts, and titles were checked cautiously to prevent duplication of datasets.

### Inclusion and exclusion criteria

Studies that were included in the present meta-analysis had to comply the following norms: (1) only case-control studies, (2) studies which focused on +936C/T polymorphism and colorectal cancer risk, (3) adequate data about allele or genotype frequencies containing +936C/T genotypes (CC, CT, and TT) which could be expressed as odds ratio (OR) and corresponding 95% confidence interval (95% CI), and (4) only studies on human subjects with full content in English. Accordingly, the following exclusion criteria were also used: (1) duplication of retrieved information; (2) studies that have only case population details, reviews, abstracts, and editorials that were published in journals; (3) studies with inadequate genotype data; (4) pharmacogenetic studies, pharmacokinetic studies, interim analysis, and case reports were excluded; and (5) if a study fails to satisfy Hardy–Weinberg equilibrium (HWE).

### Data extraction and quality assessment

Data was independently and separately collected and checked for discrepancies by two investigators (KSS and ZSS). The studies included in the meta-analysis are listed in Table 1. The data collected for the study comprised the name of the first author, year of publication, population, ethnicity, genotyping method, sample size of case and control groups, genotype distributions in case and control groups, HWE, and *p*-values for controls.

**Table 1:** Characteristics of the studies included in the meta-analysis

Authors name	Year	Technique	Ethnicity	Country	No. of cases	No. of control	Case			Control			HWE
							CC	CT	TT	CC	CT	TT	
**Yang et al.** **[**39**]**	2017	iMLDR	East Asian	China	371	246	243	118	10	180	62	4	0.74
**Ahmad et al.** **[**40**]**	2016	PCR/pyrosequencing	Caucasian	Sweden	150	150	114	29	7	101	47	2	0.17
**Jannuzzi et al.** **[**41**]**	2015	PCR/RFLP	Caucasian	Turkey	103	129	78	23	2	93	32	4	0.54
**Credidio et al.** **[**42**]**	2014	PCR/RFLP	Caucasian	Brazil	261	261	210	48	3	205	54	2	0.44
**Lau et al.** **[**43**]**	2014	TaqMan	South Asian	Malaysia	130	212	99	31	0	151	57	4	0.6
**Jang et al.** **[**44**]**	2013	Sequencing	East Asian	Korea	390	492	349	130	13	244	135	11	0.13
**Antonacopoulou et al.** **[**45**]**	2011	TaqMan	Caucasian	Greece	223	264	178	80	5	151	67	6	0.65
**Wu et al.** **[**46**]**	2011	TaqMan	East Asian	China	224	200	158	59	7	166	31	3	0.27
**Wu et al.** **[**47**]**	2009	PCR/RFLP	Caucasian	German	157	117	123	31	3	88	28	1	0.44
**Ungerbäck et al.** **[**48**]**	2009	SNuPe™ genotyping kit	Caucasian	Sweden	302	336	197	91	14	239	88	9	0.79
**Bae et al.** **[**49**]**	2008	PCR	East Asian	Korea	262	229	170	83	9	169	57	3	0.45
**Chae et al.** **[**50**]**	2008	PCR/DHPLC	East Asian	Korea	465	413	293	156	16	252	149	12	0.06
**Hofmann et al.** **[**51**]**	2008	TaqMan	Caucasian	Austria	427	427	331	88	8	308	108	11	0.67

*HWE* Hardy–Weinberg equilibrium, *iMLDR* improved multiplex ligation detection reaction, *RFLP* restriction fragment length polymorphism, *DHPLC* denaturing high-performance liquid chromatography

### Statistical analysis

Pooled odds ratio (OR) and corresponding 95% confidence interval limits were calculated for evaluating the strength of the associations between the VEGF-A +936C/T polymorphism with susceptibility to colorectal cancer. The pooled odds ratios were calculated by fixed effects model or random effects model, according to the heterogeneity level. The amount of heterogeneity was identified by performing the Cochran’s *Q*-test and the *I*^2^ test. The amount of heterogeneity was calculated according to the following scale: 75% ≥ *I*^2^ = very severe heterogeneity, 50 ≥ *I*^2^ < 75% = severe heterogeneity, 25 ≥ *I*^2^ < 50% = moderate heterogeneity, and *I*^2^ < 25% = very less heterogeneity; high-resolution forest plots were prepared to portray both OR and 95% CI limits. To assess publication bias, we examined funnel plots. As there was minimal inter-study heterogeneity, sensitivity analysis was not performed. All included studies were tested for genotypic distribution of the VEGF-A +936C/T polymorphism in the control group with the HWE principle using the chi-square goodness-of-fit test. *p* < 0.05 was considered as statistically significant. Subgroup analysis was performed to check the association between VEGF-A +936C/T polymorphism and the ethnicity of the study subjects. The analysis was conducted using a gratuitous web tool MetaGenyo [37], a web application framework for RStudio. Backend computations were carried out in R using available packages and custom scripts.

### Quality assessment of the studies

The quality of all the studies included in the meta-analysis was assessed by Newcastle-Ottawa scale (NOS) [38]. NOS is a star system which allows semiquantitative evaluation of nonrandomized study quality; it comprises of eight items which are categorized into three major components such as selection, comparability and exposure (case-control studies), or outcome (cohort studies). The scale ranges from zero to nine stars; the number of stars represented the highest methodological quality. The study is considered to be of good quality if the total score is above 5. Subgroup analysis was performed according to the quality of the study and the total score obtained.

## Results

### Meta-analysis

**Fig. 1:**
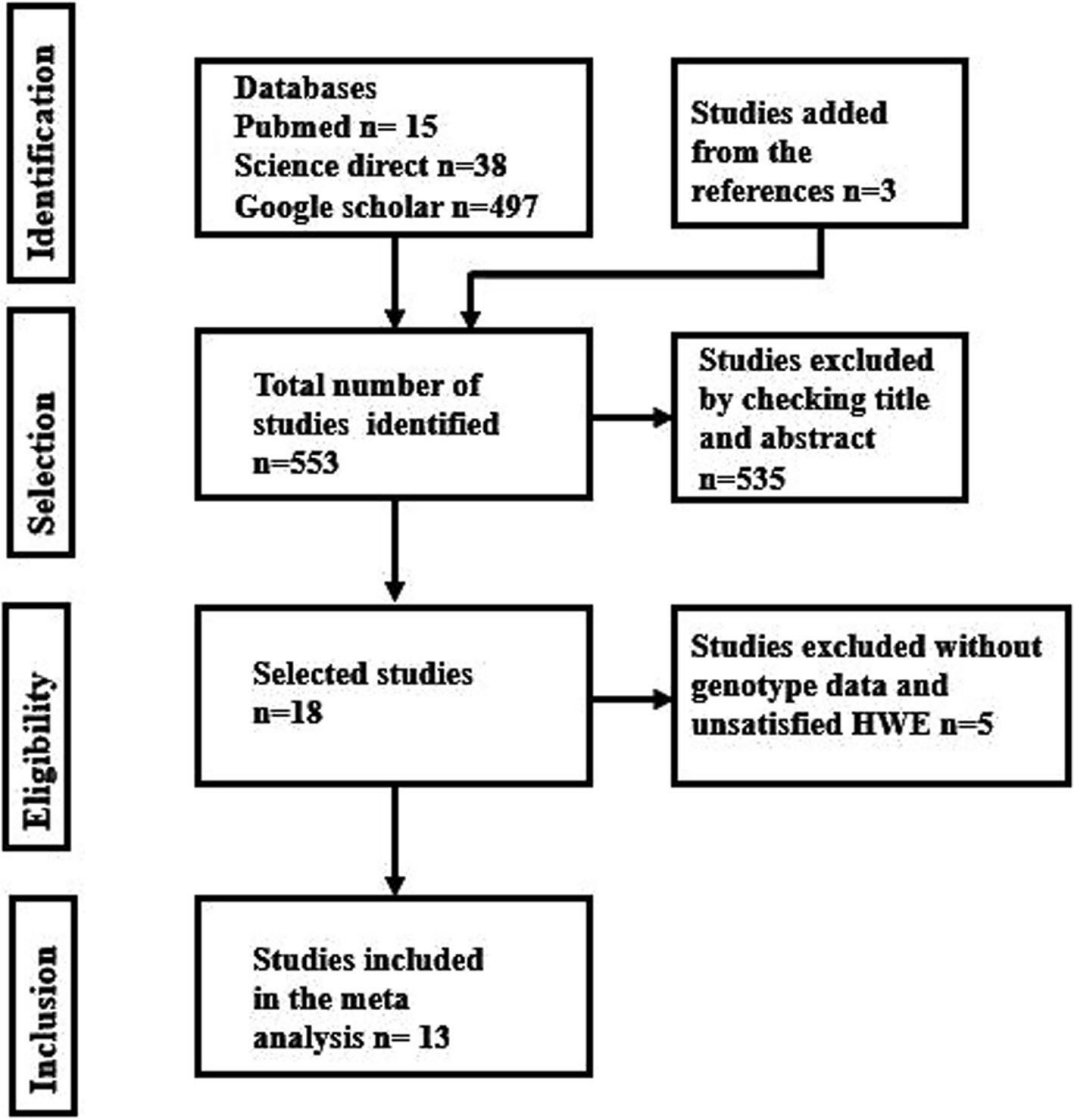
A schematic, to set forth the steps taken for the meta-analysis study

The process implemented for retrieval and selection of papers in this meta-analysis is shown in Fig. 1. The baseline characteristics of the included studies are briefed in Table 1. A total of 553 studies were retrieved after a comprehensive search in the electronic databases available, out of which 535 studies were omitted after reading the abstract as they did not match the study criteria. Of the remaining 18 studies, 2 studies did not satisfy HWE, and 3 studies had inadequate genotype data; eventually, 13 studies were included in the study [39–51]. Out of these seven belonged to Caucasian population, five belonged to East Asians, and one South Asian. The quality of studies included was assessed, and points were given on a scale of five to nine (Table 2). Thirteen studies were of good quality (Table 2).

**Table 2:** Newcastle-Ottawa Scale (NOS) for the quality assessment of the studies included

Study	Case definition adequate	Representativeness of the cases	Selection of controls	Definition of controls	Comparability based on design or analysis	analysis ascertainment of exposure	Same method of ascertainment for cases and controls	Non-response rate	Total scores
**Yang et al. 2017** **[**39**]**	★	★	★	★	★ ★	★	★	**-**	8
**Ahmad et al. 2016** **[**40**]**	★	★	★	★	★	**-**	★	**-**	6
**Jannuzzi et al. 2014** **[**41**]**	★	★	-	★	★ ★	★	★	-	7
**Credidio et al. 2014** **[**42**]**	★	★	★	★	★ ★	★	★	**-**	8
**Lau et al. 2014** **[**43**]**	★	★	★	★	★	★	★	**-**	7
**Jang et al. 2013** **[**44**]**	★	★	★	★	★	★	★	**-**	7
**Antonacopoulou et al. 2011** **[**45**]**	★	★	★	★	★	★	★	**-**	7
**Wu et al. 2011** **[**46**]**	★	★	★	★	★	★	★	**-**	7
**Wu et al. 2009** **[**47**]**	★	★	★	★	★ ★	★	★	-	8
**Ungerbäck et al. 2009** **[**48**]**	★	★	★	★	★★	★	★	**-**	8
**Bae et al. 2008** **[**49**]**	★	★	★	★	★ ★	**-**	★	**-**	7
**Chae et al. 2008** **[**50**]**	★	★	★	★	★	★	★	**-**	7
**Hofmann et al. 2008** **[**51**]**	★	★	★	★	★	★	★	**-**	7

*OR* odds ratio, *CI* confidence interval, *I*^2^ I square; each star represents the fulfilled individual criteria within the subsection

### Association between VEGF-A +936C/T polymorphism and colorectal cancer risk

**Fig. 2:**
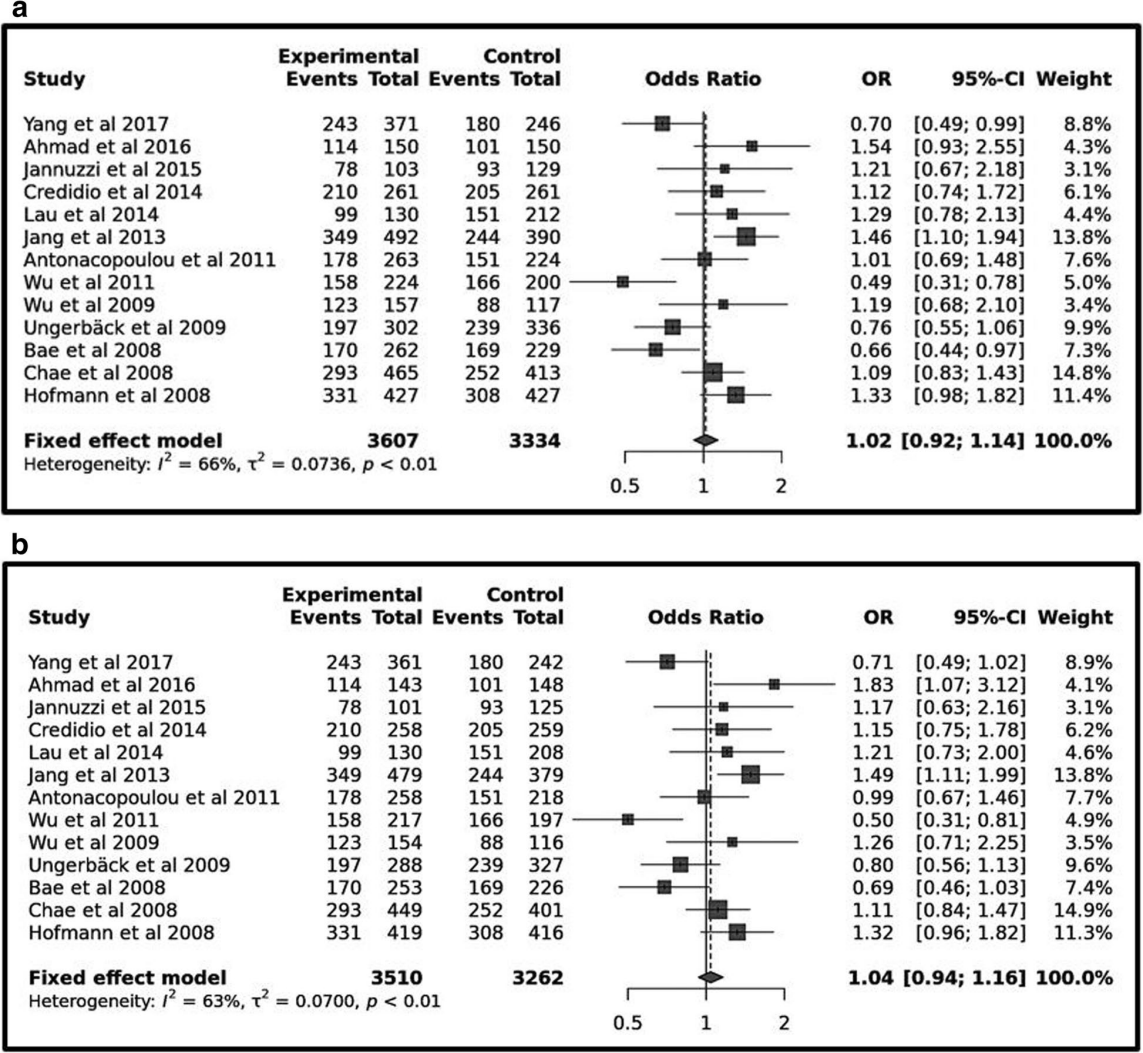
**a** Association between VEGF-A +936C/T polymorphism and colorectal cancer risk in CC vs. CT + TT model presented as a forest plot. **b** Association between VEGF-A +936C/T polymorphism and colorectal cancer risk in CC vs. CT model presented as a forest plot

**Fig. 3:**
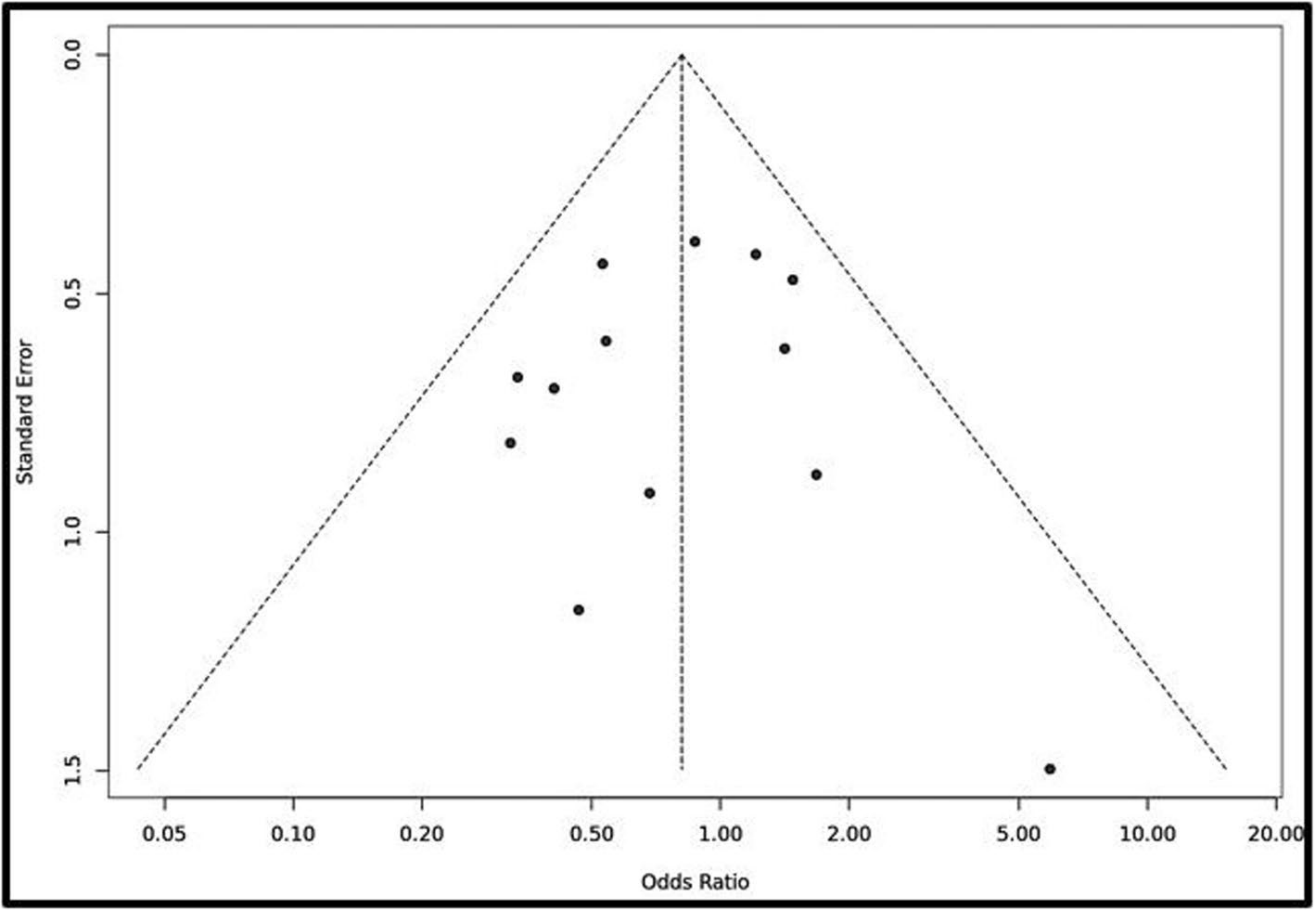
Association between VEGF-A +936C/T polymorphism and colorectal cancer risk presented as a funnel plot

Thirteen studies compared the association of VEGF-A +936C/T with colorectal cancer patients including a sample size of *n* = 3465 and healthy controls having a sample size of *n* = 3476. Fixed-effects model was used; pooled OR investigations and the results were represented as forest plots (Fig. 2 a and b). With respect to the OR and 95% CI, no significant association was observed between VEGF-A +936C/T polymorphism and colorectal cancer (Fig. 2 a and b; Table 3) in the allelic (C vs. T: *OR* = 0.99; 95% *CI* = 0.91–1.09); recessive (CC vs. CT+TT: *OR* =1.02; 95% *CI* = 0.9202–1.1352); dominant (CC + CT vs. TT: *OR* = 0.80; 95% *CI* = 0.58–1.10); overdominant (CT vs. CC + TT: *OR* = 0.95; 95% *CI* = 0.85–1.06); and the CC vs. CT : (*OR* = 1.04; 95% *CI* = 0.93–1.16) genetic models. However, the subgroup analysis revealed significant results in the overdominant model (CT vs. CC + TT: *OR =* 1.5047, 95% *CI =* 1.19–1.90) in East Asians (Table 4). In Caucasian population, no association was observed in the overdominant model (CT vs. CC + TT: *OR* = 0.8843*,* 95% *CI =* 0.74–1.04). The funnel plot of studies evaluating the role of +936C/T appeared symmetric (Fig. 3), suggesting the absence of publication bias (Egger’s test *p*-value = 0.8515). The subgroup analysis for the study quality revealed significant results in the CC vs. CT model (*OR* = 1.82, 95% *CI =* 1.07–3.12). The forest plot for the recessive model showed heterogeneity of *I*^2^ =66%, and CC vs. CT model showed *I*^2^ = 63% (Fig. 2 a and b).

**Table 3:** Association between VEGF-A +936C/T polymorphism and the risk of developing CRC in all ethnicities

Model	Test of association			Test of heterogeneity		Publication bias
Number of studies	OR	95% ***CI***	***p***-value	***p***-value	***I***^**2**^	Egger’s test ***p***-value
C vs. T	0.9983	[0.91–1.09]	0.9716	0.0007	0.6458	0.6927
CC vs. CT + TT	1.0221	[0.92–1.13]	0.6837	0.0004	0.6591	0.6999
CC + CT vs. TT	0.8024	[0.58–1.10]	0.1792	0.6291	0	0.8515
CT vs. CC + TT	0.9525	[0.85–1.06]	0.3757	0.0019	0.6143	0.7475
CC vs. TT	0.8123	[0.58–1.12]	0.2073	0.4898	0	0.8176
CC vs. CT	1.0426	[0.93–1.16]	0.4500	0.001	0.634	0.7373
CT vs. TT	0.7838	[0.56–1.09]	0.1493	0.8074	0	0.9292

*OR* odds ratio, *CI* confidence interval, *I*^2^ I square

**Table 4:** Summary of the association between VEGF-A +936C/T polymorphism and CRC in different genetic models and according to ethnicity

Models	Ethnicity and quality of the study	Parameters					
		***OR***	95% ***CI***	Association ***p***-value	Heterogeneity ***p***-value	***I***^**2**^	Egger’s test ***p***-value
**C vs. T**	**Overall**	0.9983	[0.91–1.09]	0.97169	0.0007	0.6458	0.6927
	**East Asian**	0.6537	[0.53–0.80]	0.00003	0.4693	0	0.0177
	**South Asian**	1.3375	[0.84–2.11]	0.2141	NA	NA	NA
	**Caucasian**	1.0563	[0.91–1.22]	0.4637	0.1745	0.3493	0.6018
	**Quality of the study**	1.2242	[0.78–1.90]	0.3694	NA	NA	NA
**CC vs. CT + TT**	**Overall**	1.0221	[0.92–1.13]	0.68371	0.0004	0.6591	0.6999
	**East Asian**	0.6273	[0.49–0.78]	0.00006	0.4845	0	0.1031
	**South Asian**	1.2901	[0.78–2.12]	0.3191	NA	NA	NA
	**Caucasian**	1.0982	[0.92–1.29]	0.27	0.1489	0.3855	0.5338
	**Quality of the study**	1.5363	[0.92–2.55]	0.0967	NA	NA	NA
**CC + CT vs. TT**	**Overall**	0.8024	[0.58–1.10]	0.17928	0.6291	0	0.8515
	**East Asian**	0.4813	[0.23–1.00]	0.0518	0.8723	0	0.4451
	**South Asian**	5.6331	[0.3008; 105.4861]	0.2475	NA	NA	NA
	**Caucasian**	0.8538	[0.52–1.39]	0.5264	0.3652	0.08	0.7439
	**Quality of the study**	0.2761	[0.05–1.35]	0.1121	NA	NA	NA
**CT vs. CC + TT**	**Overall**	0.9525	[0.85–1.06]	0.37577	0.0019	0.6143	0.7475
	**East Asian**	*1.5047*	*[1.19–1.90]*	*0.0006*	0.4893	0	0.1881
	**South Asian**	0.8515	[0.51–1.41]	0.5325	NA	NA	NA
	**Caucasian**	0.8843	[0.74–1.04]	0.1603	0.1346	0.4061	0.4246
	**Quality of the study**	0.5252	[0.30–0.89]	0.0177	NA	NA	NA
**CC vs. CT**	**Overall**	1.0426	[0.93–1.16]	0.45	0.001	0.634	0.7373
	**East Asian**	0.6478	[0.51–0.81]	0.0002	0.4897	0	0.1603
	**South Asian**	1.2055	[0.72–1.99]	0.46867	NA	NA	NA
	**Caucasian**	1.1245	[0.94–1.33]	0.1824	0.1323	0.4095	0.4483
	**Quality of the study**	*1.8293*	*[1.07–3.12]*	*0.0268*	NA	NA	NA

*OR* odds ratio, *CI* confidence interval, *I*^2^ I square.*Italicized values are statistically significant

## Discussion

VEGF is an imperative regulator of tumor angiogenesis, associated with the development and progression of multiple cancers [52]. This gene portrays a pivotal role and acts as an important prognostic factor in a variety of tumors, including CRC. Numerous studies related to the risk and diagnosis of breast cancer [53] and non-small cell lung cancer [54] have demonstrated their impact on VEGF SNPs. Several studies have also revealed that VEGF protein production during colorectal carcinogenesis is correlated with polymorphisms situated in the 5′ and 3′ UTR of the VEGF gene and their promoter region [13, 55]. However, very limited studies have reported about the association of VEGF-A 3′ UTR miRNA SNPs with the susceptibility to CRC.

MiR-199a is an intronic miRNA discovered in 2003 and associated with development of various diseases [56]. It has been reported that MiR-199a is a possible inhibitor of HIF-1α/VEGF pathway. MiR-199a targets the 3′ UTR of HIF-1α and HIF-2α and leads to the decrease in hypoxia-increased HIF levels. HIF-1α is an important transcription factor, which plays a crucial role in CRC development and progression [57]. Overexpression of VEGF gene in CRC cells is observed often; this gene portrays a vital role in angiogenesis and cell proliferation which makes it a potential target for cancer therapy. The mechanism involved in VEGF-A 3′ UTR binding site polymorphism regulating the development and progression of CRC remains scanty. Currently, there are very few studies related to miR199a significance in angiogenesis and colorectal cancer. Hence, we wanted to elucidate the contribution of VEGF-A 3′ UTR binding site polymorphism to colorectal cancer by performing a meta-analysis.

The 3′ UTR of the *VEGF* gene has demonstrated to enhance the stability of mRNA and also leads to the hypoxic induction of the VEGF gene [18, 58, 59]. Recently, genes designated as Hu family have been ascertained, and their products have shown to bind to the AU-rich element of 3′ UTR of numerous genes, including the *VEGF* mRNA [18, 60]. It is proposed that the proteins of the Hu family change the *VEGF* mRNA conformation so that the mRNA is not affected by RNase. SNPs in the 3′ UTR have shown to be related with the deregulation of the affected genes [61]. Therefore, the SNPs in the 3′ UTR may modify the mRNA conformational integrity, bringing about genetic variation of *VEGF* gene expression. The VEGF +936 C/T is a significant functional polymorphism that has demonstrated to alter the susceptibility of various diseases such as cancer [62, 63]. Chen et al. stated that the total viability of non-small cell lung carcinoma patients and their response to chemotherapy was afflicted of VEGF +936C/T gene polymorphism [55]. Zhang et al. reported that VEGF +936C/T was significantly correlated with glioma susceptibility and may act as a genetic marker [64]. VEGF +936C/T serves as a marker for disease aggression, relapse, and an important factor for the poor prognosis of epithelial ovarian cancer [65].

Most of the studies reported lack of association between CRC and +936C/T in Caucasian population [47, 51, 66]. These results were consistent with studies recently published in the Caucasian population [40, 41]. Our meta-analysis showed concordance to these results since no association was observed in Caucasian population (Table 4). However, the +936T allele increases the risk of CRC in East Asian population such as Korean and Chinese populations [47, 49]. Similarly, Jang et al. reported that the +936T allele was associated with an increased susceptibility to CRC [44, 67]. These results were concordant to the results obtained in our meta-analysis (Table 4).

Jeon et al. study dealing with the association amidst VEGF 3′ UTR polymorphisms and CRC susceptibility in Koreans stated that VEGF 1451C *>* T and 1725G *>* A could render to CRC susceptibility [68]. Also, metastasis and angiogenesis are eminently connected to VEGF expression in solid tumors. VEGF +936-T allele leads to reduced plasma VEGF levels in young healthy population of Caucasians [69]; on the other hand, the underlying mechanism remains to be elusive. Two interpretations were put forth; (1) +936 C/T mutation induces the loss of a potential binding site for AP-4, which is a transcription factor enhancing expression of several viral and cellular genes by binding to specific enhancer sites [70, 71]. (2) This variant may hinder the binding of hypoxia-induced protein to the 3′ UTR of VEGF-A mRNA, which may lead to a significantly diminished half-life of the mRNA.

Subsequently, the polymorphisms in VEGF-A gene 3′ UTR can alter the implied binding sites of transcription factors, which stimulate impaired proteins and malformations. This malformation could illustrate why the +936-T allele carriers have a lower risk of developing cancer like breast cancer, small cell lung cancer, and oral squamous cell carcinoma [72, 73]. Lack of association was observed in VEGF-A +936 C/T polymorphism and CRC in Han Chinese in Sichuan province subsequent to Bonferroni correction [39]. Studies in large numbers are requisite to directly check for miRNA binding activity to VEGF-A 3′ UTR polymorphisms and to regulate the mechanism by which these polymorphisms might have an effect on cellular proliferation and cancer advancement. These studies might have a great clinical impact for all diseases associated with abnormal angiogenesis and hypoxic conditions.

Majority of studies reported a lack of association between CRC and VEGF-A +936C/T polymorphism. Lately, few case-control studies have been carried out to examine the association between VEGF gene polymorphisms and CRC susceptibility [39–41]. However, the possible effect of VEGF-A +936C/T polymorphisms on VEGF-A production as well as tumor development and progression in CRC still remains ambiguous. Hence, the purpose of this study was to investigate the effect of VEGF-A +936C/T polymorphisms on susceptibility to CRC by means of a meta-analysis.

The current meta-analysis included 13 case-control studies, which consists of 3465 CRC cases and 3476 controls. Our meta-analysis suggests that the VEGF-A +936C/T gene polymorphism is not associated with the risk of developing CRC. In this investigation, we did not find any evidence of publication bias as shown in Fig. 3. VEGF-A gene polymorphisms have been associated with susceptibility to several cancer types. Previously, two meta-analyses have been performed on −460T/C, −634G/C, +936C/T, −2578C/A, −1154G/A, and + 405C/G [14, 74]. These two meta-analyses indicated that VEGF +936C/T demonstrated no association with colorectal cancer. Our updated meta-analysis results show association among East Asian population which shows concordance to meta-analysis conducted by Gholami et al. [75]. From our study, we have also observed that in the CT vs. CC+TT, OR is 1.5 for East Asians. When compared with the overall pooled odds ratio for the overdominant model (*OR* = 0.9525; *p*-value = 0.37577), the East Asian subgroup exhibits an increase in the odds ratio indicating that the SNPs effect varies with ethnicity. Several studies have focused on VEGF-A +936C/T polymorphism for its possible association with colorectal cancer patients. This is the first meta-analysis to be solely conducted on VEGF-A miRNA binding site polymorphism +936C/T in colorectal cancer.

Our study has few possible limitations. (a) Only published studies were included in the meta-analysis, (b) only three databases were searched for relevant articles, (c) Lack of genotype frequency information provided by some published studies, (d) HWE not getting satisfied did not allow the estimation of the best genetic model of inheritance to follow, (e) all case-control studies were obtained from Asian and Caucasian population, (f) and gene-gene and gene-environment interactions were not accounted. Finally, although all cases and controls of each study were explicitly defined with similar inclusion criteria, there may be some potential factors that were not taken into account that may have influenced our results.

## Conclusions

To conclude, this meta-analysis suggests that the VEGF-A +936C/T gene polymorphism is not associated with the risk of developing CRC in Caucasian and South Asian population. But the minor allele present in the East Asian population was related to an increased risk of developing CRC. Based on our findings, additional larger population-based studies with diverse ethnic groups are imperative to validate the association of VEGF-A gene polymorphisms and colorectal cancer.

## Data Availability

Not applicable.

## Abbreviations

VEGF-A: Vascular endothelial growth factor A
UTR: Untranslated region
MiRNA: MicroRNA
mRNA: Messenger RNA
CRC: Colorectal cancer
HIF-1α: Hypoxia-inducible factor 1-alpha
NOS: Newcastle-Ottawa scale
HWE: Hardy–Weinberg equilibrium
OR: Odds ratio
CI: Confidence interval
